# Emotional Expression in Simple Line Drawings of a Robot's Face Leads to Higher Offers in the Ultimatum Game

**DOI:** 10.3389/fpsyg.2017.00724

**Published:** 2017-05-22

**Authors:** Kazunori Terada, Chikara Takeuchi

**Affiliations:** Informatics Course, Department of Electrical, Electronics and Computer Engineering, Faculty of Engineering, Gifu UniversityGifu, Japan

**Keywords:** robot, facial expression, emotion, altruistic behavior, human-robot interaction

## Abstract

In the present study, we investigated whether expressing emotional states using a simple line drawing to represent a robot's face can serve to elicit altruistic behavior from humans. An experimental investigation was conducted in which human participants interacted with a humanoid robot whose facial expression was shown on an LCD monitor that was mounted as its head (Study 1). Participants were asked to play the ultimatum game, which is usually used to measure human altruistic behavior. All participants were assigned to be the proposer and were instructed to decide their offer within 1 min by controlling a slider bar. The corners of the robot's mouth, as indicated by the line drawing, simply moved upward, or downward depending on the position of the slider bar. The results suggest that the change in the facial expression depicted by a simple line drawing of a face significantly affected the participant's final offer in the ultimatum game. The offers were increased by 13% when subjects were shown contingent changes of facial expression. The results were compared with an experiment in a teleoperation setting in which participants interacted with another person through a computer display showing the same line drawings used in Study 1 (Study 2). The results showed that offers were 15% higher if participants were shown a contingent facial expression change. Together, Studies 1 and 2 indicate that emotional expression in simple line drawings of a robot's face elicits the same higher offer from humans as a human telepresence does.

## 1. Introduction

Recently, there has been increasing interest and progress in robotic emotional expressions. A wide variety of methods for achieving emotional expression have been proposed (Bethel and Murphy, 2008), including facial expressions (Bartneck, 2003; Breazeal, 2004; Kanoh et al., 2004; Itoh et al., 2006; Matsui et al., 2010), speech (Kim et al., 2009a,b), body movement (Shimokawa and Sawaragi, 2001; Bethel and Murphy, 2007), and colors (Sugano and Ogata, 1996; Kim et al., 2009a,b; Terada et al., 2012). Leaving aside discussion regarding a robot's ability to possess genuine emotions, implementing a display of emotion in robots could be useful not only by increasing their friendliness but also by helping them to influence people without explicit language (Breazeal, 2003, 2004).

There have been studies on the effect of robotic emotions on human behavior (Cassell and Thorisson, 1999; Bickmore and Picard, 2005; Leyzberg et al., 2011); these focused on the task-oriented effects of emotions. Leyzberg et al. (2011) showed that robots that express emotions elicited better human teaching. A long-term experiment conducted by Bickmore and Picard (2005) showed that an agent with relational behavior, including social-emotional responses, contributed to increasing participants' positive attitude about exercise. While these studies revealed that robots with emotions positively affect human behavior, the nature, and essential function of these emotions have not been discussed. In the present study, we focused on the social functional aspect of emotions and experimentally investigated the effect of emotional expression as depicted through a simple line drawing of a face on human economic behavior.

Emotions control the behavior of an agent. For example, fear increases heart rate and muscle tension and drives an agent to escape from a situation; consequently, fear helps in avoiding dangerous situations. Emotions affect not only one's own behavior but also that of others. An angry individual, for example, usually obtains concessions from a competitor in a conflict situation (van Kleef et al., 2004; Sinaceur and Tiedens, 2006; van Kleef and Côté, 2007; van Dijk et al., 2008; van Kleef et al., 2008; Sell et al., 2009; Fabiansson and Denson, 2012; Reed et al., 2014). Positive emotions are considered to have evolved to maintain cooperative relationships (Trivers, 1971; Alexander, 1987; Frank, 1988; Scharlemann et al., 2001; Brown and Moore, 2002; Brown et al., 2003; Mehu et al., 2007; Reed et al., 2012; Mussel et al., 2013).

Altruism is a behavior that reduces the actor's wealth while increasing the wealth of the recipient, whereas cooperation is a process in which agents work together to gain common or mutual wealth. However, altruism can be considered to be asynchronous cooperative behavior by considering direct or indirect reciprocity (Nowak and Sigmund, 2005). In order to produce altruistic behavior, one must ignore the loss of one's own wealth. Positive emotions such as happiness and kindness that are elicited from another's facial expressions presumably compensate for the loss. Therefore, emotion is more important for long-term or indirect reciprocal relationships than short-term (one-shot) cooperative tasks. We used altruistic behavior as a measure of the function of the robot's facial expression because our focus is on the long-term human-robot relationship.

Researchers have been investigating whether people have a tendency to cooperate with robots (Nishio et al., 2012; Torta et al., 2013; Sandoval et al., 2016). Decision making in economic games such as the prisoners' dilemma and the ultimatum game is used to measure the cooperative attitude of participants. Nishio et al. (2012) have studied how the appearance of agents (computer, humanoid, android, or human) affects participants' cooperativeness. They conclude that although the appearance of agents does not affect cooperativeness, conversation with a human-like agent (android) leads people to be more cooperative. Torta et al. (2013) reported that rejection scores in the ultimatum game are higher in the case of a computer opponent than in the case of a human or robotic opponent, indicating that people might treat a robot as a reciprocal partner. Sandoval et al. (2016) showed that participants who interacted with a robot showed significantly less cooperation than when they interacted with a human in the prisoner's dilemma. Further, participants offered significantly less money in the ultimatum game to the robot than to the human agent, indicating that people tend to cooperate more with a human agent than with a robot.

From the above discussion, the following prediction could be derived: if robots offer emotional expression, people behave more cooperatively toward them. There are a few studies that examine the effect of the emotional expression of robots on human cooperative behavior in terms of economic behavior (de Melo et al., 2010, 2011). de Melo et al. (2010) conducted an experiment in which participants play the iterated prisoner's dilemma against two different virtual agents that play the tit-for-tat strategy but communicate different goal orientations (cooperative vs. individualistic) through their patterns of facial displays. They showed that participants were sensitive to differences in the facial displays and cooperated significantly more with the cooperative agent. de Melo et al. (2011), in another study, reported that participants conceded more to a virtual agent that expresses anger than to one that expresses happiness in a negotiation task.

The studies of de Melo et al. (2010, 2011) used human-like virtual character agents. In our study, we used a real robot with a simple line drawing of a face to remove realistic and biological human features from the agent's face (Terada et al., 2013). Most of the robots that are used in human-robot interaction studies have sophisticated facial expression mechanisms (Breazeal, 2004; Itoh et al., 2006; Matsui et al., 2010; Becker-Asano and Ishiguro, 2011; Mazzei et al., 2011). The underlying assumption is that mimicking real human facial expressions induces humans to emotionally respond as they would when interacting with a real human. However, studies have revealed that line drawing facial expressions are recognized to the same extent as a realistic face (Katsikitis, 1997; Britton et al., 2008), affect human altruistic behavior even they are slightly different (Brown and Moore, 2002), and are processed in the human brain in the same way as a human face (Britton et al., 2008).

In the present study, we investigated whether a simple line drawing of a face is useful in human-robot interaction in terms of human-robot cooperative relationships. Terada et al. (2013) have showed that emotional expression by robots led people to behave more altruistically toward the robots even though the emotion was represented by simple line drawings. However, it is unclear whether this effect is the same extent as that of human-human interaction. In the present paper, we first show the results of human-robot condition reported in Terada et al. (2013) as Study 1. We then show the results of human-human condition (Study 2) and compare the results of these two studies.

The ultimatum game has been used to measure human altruistic behavior (Güth and Tietz, 1990; Sanfey et al., 2003; Oosterbeek et al., 2004; Xiao and Houser, 2005; van Dijk et al., 2008; Yamagishi et al., 2009). It is played by two players, a proposer and a responder, who are given the opportunity to split an allotment of money. The proposer has the right to divide the money and offer an amount to the responder. If the responder accepts the proposal, both players keep the money. If the responder rejects the proposal, neither player receives the money. The findings of a meta-analysis of 37 papers with 75 results from ultimatum game experiments showed that on average, the proposer offers 40% of the money to the responder, and 16% of the offers are rejected (Oosterbeek et al., 2004).

In our study, all participants were assigned to be the proposer and were instructed to decide their offer within 1 min by controlling a slider bar. In the decision period, a change in the responder's facial expression was shown to the proposer (only in the change of facial expression condition), which is not a normal procedure in the ultimatum game. The communication before the decision is treated as cheap talk, which is costless and unverifiable preplay statements about private information and non-credible threats about future actions (Croson et al., 2003). Croson et al. (2003) showed that threats of future actions influenced bargaining outcomes.

The goal of the present study was to explore whether communication using the facial expression of robots is effective in establishing human-robot cooperative relationships. We used the offer in the ultimatum game as the measurement of cooperative attitude of human toward a robot. As a result, the effectiveness of facial expression of robots in human-robot cooperative relationship could be evaluated in terms of economic value.

Studies 1 and 2 were both conducted in accordance with the recommendations of the Ethical Guidelines for Medical and Health Research Involving Human Subjects provided by the Ministry of Education, Culture, Sports, Science, and Technology and the Ministry of Health, Labor, and Welfare in Japan with written informed consent from all subjects. All subjects gave written informed consent in accordance with the Declaration of Helsinki. The protocol was approved by the Medical Review Board of Gifu University Graduate School of Medicine.

## 2. Study 1

### 2.1. Method

#### 2.1.1. Participants

Twenty-six healthy graduate and undergraduate students (15 male, 11 female, *M*_age_ = 19.62 years, *SD*_age_ = 3.85 years, age range: 18–24 years) participated in the experiment. Participants were recruited through advertising on posters and via e-mail at the university. They were informed that they would be paid with a JPY 500 (approximately USD 5) book coupon for their time. All were ignorant of the purpose of the experiment.

#### 2.1.2. Experimental design

A single-factor two-level between-participants experimental design was used. Participants were randomly assigned to either a “change of facial expression” or a “static face” condition. All participants assumed the role of the proposer and were asked to determine their offer within 1 min by controlling the slider bar. The only difference between the two conditions was whether the corners of the mouth of the line drawing shown on an LCD monitor mounted on the robot moved upward or downward according to the position of the slider bar. In the initial state, a straight line segment represented the line drawing mouth.

#### 2.1.3. Apparatus

The ultimatum game was played once. The proposer was given 100 points, which corresponds to JPY 1000 (approximately USD 10), as the amount to divide. The proposer was given 1 min to determine the offer (*decision phase*). During the decision phase, the proposer adjusted the offer by controlling the slider bar. Participants were informed that the game would be played against a humanoid robot that might react to the participant's offer through an LCD monitor mounted on the robot.

**Figure 1:**
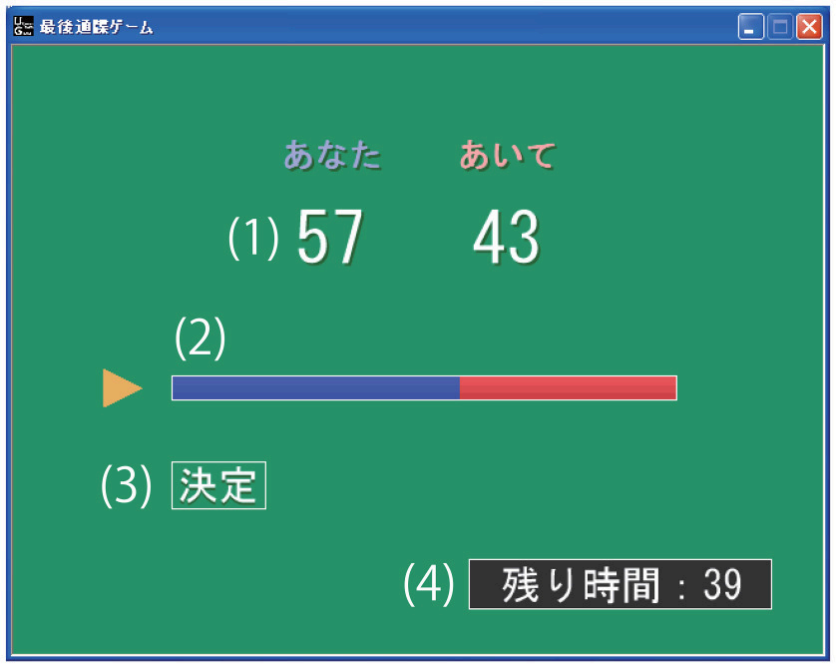
**Graphical user interface used by the proposer to determine the offer: (1) numerical representation of the offer, (2) slider bar to change the offer, (3) button for final decision, and (4) time remaining**.

A GUI was used to determine the offer and to communicate the emotional state of the responder (see Figure 1). The proposer was asked to decide the offer within 1 min by moving a slider bar on the GUI, which was controlled by a gamepad connected to the computer.

#### Static face condition

The line drawing face did not change during the proposal period.

#### Change of facial expression condition

The corners of the mouth of the line drawing moved upward and downward according to the position of and one second after the movement of the slider bar. This delay was inserted to prevent the participants from assuming that the responder was merely a simple computer program; an immediate mouth movement completely contingent on the proposer's action might strongly indicate artificiality. The software's calculation rate was 60 fps, the same as the monitor used to display the GUI.

**Figure 2:**
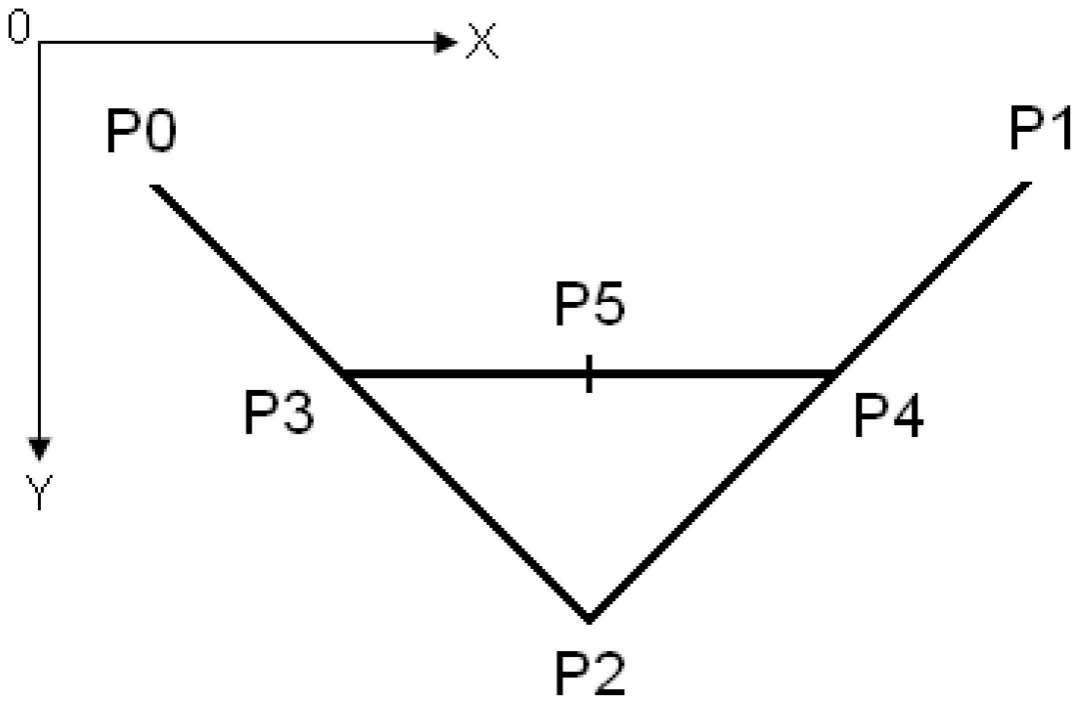
**Control points of the Bézier curve used to represent the mouth**.

**Figure 3:**
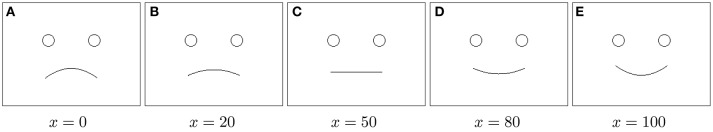
**Examples of the facial expressions displayed to the proposer as a function of the proposer's offer *x* ∈ [0, 100]**. **(A)** *x* = 0. **(B)** *x* = 20. **(C)** *x* = 50. **(D)** *x* = 80. **(E)** *x* = 100.

Figure 2 shows the control points of a Bézier curve, which represented the line drawing of the mouth. The points P3 and P4 are the static points. The Y-coordinates of the points P0, P1, and P2 changed according to the position of the slider bar. Figure 3 illustrates examples of the facial expressions shown to the proposer as a function of the proposer's offer *x* ∈ [0, 100]. If the slider bar moved to the right, the offer decreased and negative facial expressions, such as those shown in Figures 3A,B, were displayed.

**Figure 4:**
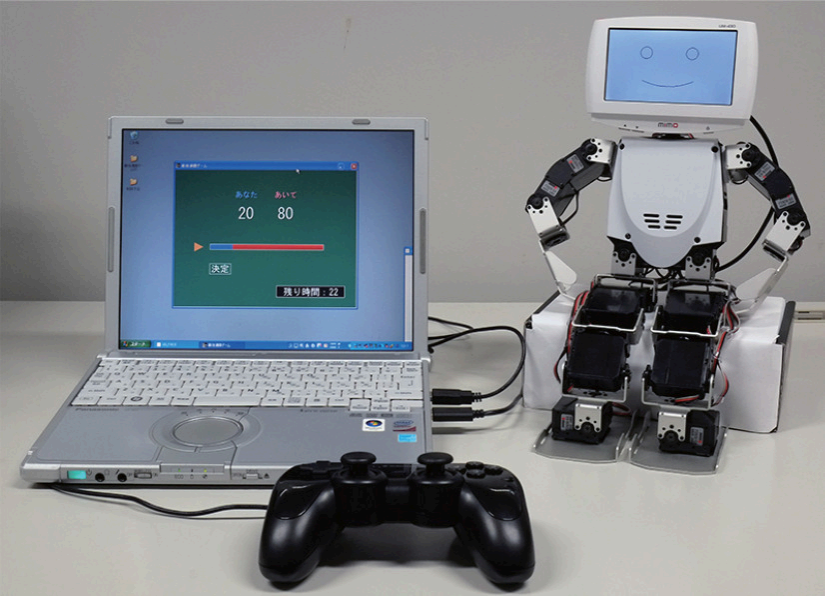
**System used in our experiment**.

Figure 4 shows the experimental system. We mounted an LCD monitor on a Robovie-X, a commercially available robot. Line drawings of facial expressions were shown on the mounted LCD monitor, which was connected to a laptop computer via a USB cable. The laptop computer was also used to display the GUI, and a gamepad for controlling the slider bar on the GUI was connected to the laptop.

#### 2.1.4. Procedure

In the experiment room, participants were asked to read an instruction sheet that stated the rules of the ultimatum game, how to use the interface, and that “the response of the responder will be shown on the head display.” In addition, they were informed that they were assigned to be the proposer and that they would win additional money according to their score in the game.

After the proposal, the participants were not immediately informed of the responder's acceptance/rejection: they were first asked to complete a questionnaire to avoid the questionnaire responses being affected by the responder's decision. After completing the questionnaire, the participants were informed that they had all played as proposers against a computer program, and they were paid with an additional JPY 500 (approximately USD 5) book coupon, the amount of money that would be given if a 50:50 offer was accepted.

#### 2.1.5. Measurement and analysis

The offer was recorded every 0.5 s. After the game, participants were asked to answer four 7-point Likert scale questions (0 = “definitely no” to 7 = “definitely yes”):

- Q1. Did you perceive emotions in the picture shown on the head of the robot?
- Q2. Did you consider the responder's emotions when deciding your offer?

After answering the post-questionnaires, participants were asked whether they realized that they had been playing against a computer program.

The one-way analysis of variance (ANOVA) was used if the data were normally and homogeneously distributed. The Welch's ANOVA was used if the data were normally distributed, but the assumption of homogeneity of variance was violated. The Mann–Whitney *U*-test was used if the data were homogeneously distributed, but the assumption of normality was rejected. The Brunner–Munzel test was used if both the assumption of normality and the homogeneity of variance were violated.

### 2.2. Results

The mean durations for deciding an offer were 28.31 s (*SD* = 17.27) and 18.69 s (*SD* = 14.26) in the change of facial expression and static face conditions, respectively. The one-way ANOVA, *F*_(1, 24)_ = 2.40, *p* = 0.13, indicated that the difference was not statistically significant.

**Figure 5:**
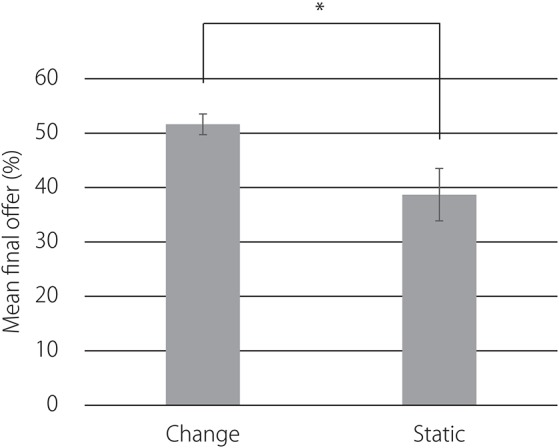
**Mean final offers over participants in both conditions**. Error bars indicate standard errors. ^*^p < 0.05.

Figure 5 presents the mean final offers over participants in both conditions. Welch's ANOVA, *F*_(1, 15.71)_ = 6.22, *p* < 0.05, showed that offers were higher in the change of facial expression condition (*M* = 51.62, *SD* = 6.91) than in the static face condition (*M* = 38.69, *SD* = 17.35).

**Figure 6:**
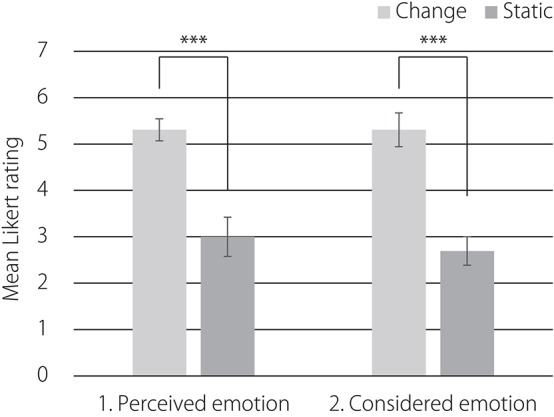
**Post-experiment questionnaire**. Error bars indicate standard errors. ^***^p < 0.001.

Figure 6 displays the results of the post-experiment questionnaire. The Mann–Whitney *U*-tests, *U* = 18.5, *z* = 3.46, *p* < 0.001, revealed that ratings for perceiving emotions from the line drawing were significantly higher in the change of facial expression condition than in the static face condition. The one-way ANOVA, *F*_(1, 24)_ = 30.03, *p* < 0.001, revealed that ratings for the consideration of emotions were significantly higher in the change of facial expression condition than in the static face condition.

Ten out of 13 participants in the change of facial expression condition realized that they had played against a computer program that generates a simple mouth movement completely contingent on the participants' action.

### 2.3. Discussion

The results show that offers were higher in the change of facial expression condition than in the static face condition, confirming that emotional expression by robots led participants to behave more altruistically toward the robots even though the emotion was represented by simple line drawings. The results of the post-experiment questionnaires support the behavioral result that the 12.92% gap between the two conditions was caused by the emotions that participants recognized from the change of facial expressions exhibited by the line drawing. Participants in the change of facial expression condition gave higher ratings, an average of 5.30, to the question “Did you perceive emotions in the picture located on the upper right of the GUI?” than did participants in the static face condition. There was a large gap, an average of 2.30, in the Q1 rating between the two conditions, which indicates that perceiving emotions caused the participants' altruistic behavior.

The conditions differed only in whether the corners of the mouth of the line drawing in the GUI changed. However, we did not explicitly inform participants that the line drawing symbolized a face or that the position of the bar represented the position of the corners of the mouth. The participants arbitrarily attributed a facial property to the geometric line drawings and attributed emotions to variable Bézier curves. According to Ekman (2003), a convex mouth shape, in which the corners of the lips curl downward, indicates sadness, and a concave mouth shape, in which corners of the lips move upward, indicates happiness. Although, we did not identify the emotions that participants perceived from the line drawings, the universality of facial expressions supports the assumption that participants recognized sadness when they were shown a convex mouth and happiness when they were shown a concave mouth.

Our results show that although a substantial number of participants (78%) in the change of facial expression condition realized that the mouth movement was controlled by a computer program, the effect of facial expression was still observed. de Melo et al. (2011) reported similar findings from a study in which participants were involved in a negotiation with computer agents. Taken together, these findings imply that facial expressions are effective in inducing people to cooperate with robots even though they know that the expressions are controlled by a program.

A meta-analysis of 75 results from ultimatum game experiments revealed that the proposer usually offers 40% of the money to the responder (Oosterbeek et al., 2004). However, participants in the change of facial expression condition offered an average of 51.62% of the money. This indicates that the offer increased by approximately 10% when people were shown changes of facial expression corresponding to their offer. By contrast, participants in the static face condition offered an average of 38.69%. This value roughly corresponds to that offered in the earlier studies that included no emotional interaction in their experimental setting.

There are two potential reasons why participants in the change of facial expression condition offered approximately 50:50, which is a fair offer. The first is the impression that the responder has the capability to respond emotionally, which is formed by the dynamic change of facial expression in response to the participant's operation. In this case, the facial expression itself does not have an absolute meaning: simply perceiving adaptivity or the ability to respond to the user's input might be lead to a fair offer. The second reason is a neutral face. In our experimental setting, a neutral face, in which the mouth was represented by a straight line, was displayed to participants when the offer was 50%. Participants could adjust the slider bar to make the facial expression neutral. Further investigation, in which a neutral face does not correspond to a 50% offer, is needed to test these two hypotheses.

Our results do not identify whether positive or negative emotion contributed to an increase in the offer. It is known that expressing anger can elicit concessions from others (van Kleef et al., 2004; Sinaceur and Tiedens, 2006; van Kleef and Côté, 2007; van Dijk et al., 2008; van Kleef et al., 2008; Sell et al., 2009; Fabiansson and Denson, 2012; Reed et al., 2014), while happiness can elicit altruism (Scharlemann et al., 2001; Brown and Moore, 2002; Brown et al., 2003; Mehu et al., 2007; Mussel et al., 2013). These findings suggest that both the negative and positive expressions shown in our experiment might have contributed to the proposer raising the offer.

Croson et al. (2003) showed that threats of future actions influenced bargaining outcomes. The negative emotional expression that was contingently presented when a low offer was proposed might have played the role of cheap talk.

## 3. Study 2

Study 2 was conducted to compare the result of Study 1 with those from a study in which participants played the game against a human responder in a teleoperation setting through a computer display. The aim of this study was to determine whether the altruistic behavior induced by the robot's facial expression is also induced by a facial expression controlled by a human.

### 3.1. Method

#### 3.1.1. Participants and experimental design

Forty healthy graduate and undergraduate students (35 male, 5 female, *M*_*age*_ = 21.38 years, *SD*_*age*_ = 1.51 years, age range: 18–23 years) participated in the experiment. All participants were ignorant of the purpose of the experiment.

As in Study 1, a single-factor two-level between-participants experimental design was used. Participants were randomly assigned to either a “static face” or a “change of facial expression” condition.

#### 3.1.2. Apparatus

**Figure 7:**
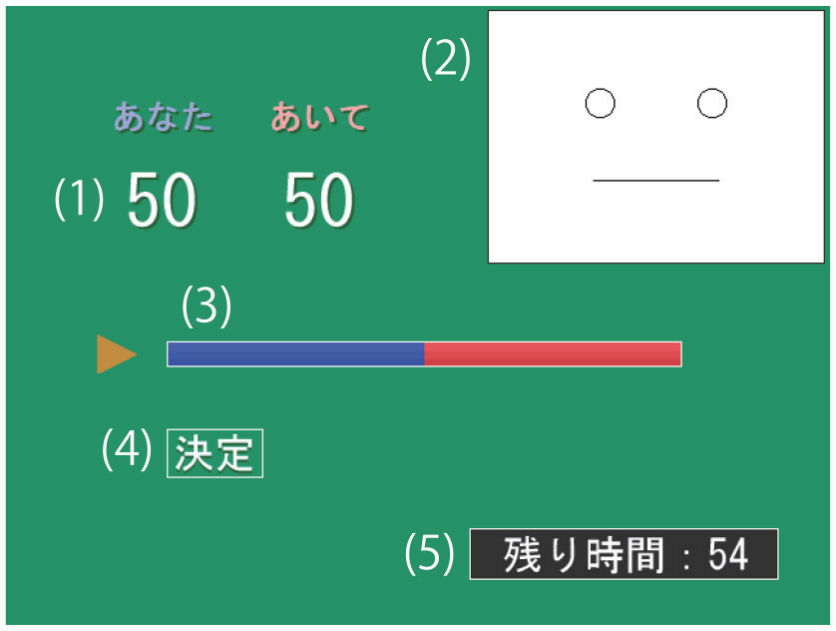
**Graphical user interface used in Study 2**.

The apparatus used was identical to that used in Study 1 except that the facial expression was shown on the upper right area of the GUI as shown in Figure 7.

#### 3.1.3. Procedure

The procedure was identical to that used in Study 1, except for the following changes. The experiment was conducted on two participants who knew each other. The two participants came to the experiment together and were taken to different rooms. In their different rooms, they were asked to read the instruction paper, and *both participants*were informed that they were assigned to be the *proposer*. Thus, all participants played the role of the proposer without knowing it. They were informed that “the response of your partner will be shown on the upper right area of the interface.” The facial expression was automatically changed based on the position of the slider bar controlled by the participant, as in Study 1.

### 3.2. Results

The data of one participant in each of the two conditions were excluded because they reported that they realized that they were playing against a computer program.

The mean durations spent deciding the amount of the offer were 50.31 s (*SD* = 12.55) and 46.47 s (*SD* = 14.68) in the facial expression change condition and static face condition, respectively. The Mann–Whitney *U*-tests, *U* = 162, *z* = 0.54 *p* = 0.59, show that no statistically significant difference was observed.

**Figure 8:**
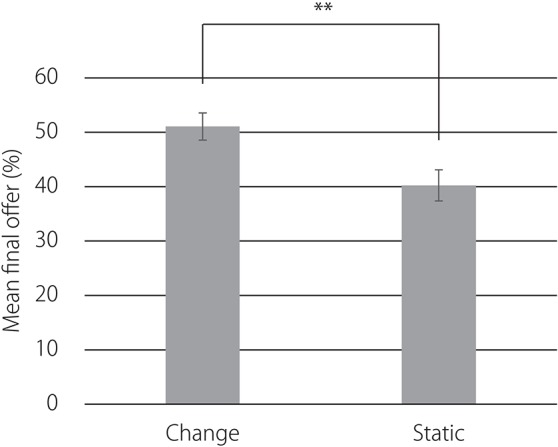
**Mean final offers averaged over participants in each of the two conditions**. Error bars indicate standard errors. ^**^p < 0.01.

Figure 8 presents the mean final offers averaged over participants in each of the two conditions. Error bars indicate standard errors of the mean value. The Mann–Whitney *U*-tests, *U* = 92, *z* = 2.61, *p* < 0.01, show that offers were higher in the facial expression change condition (*M* = 51.05, *SD* = 10.88) than in the static face condition (*M* = 40.21, *SD* = 12.48).

**Figure 9:**
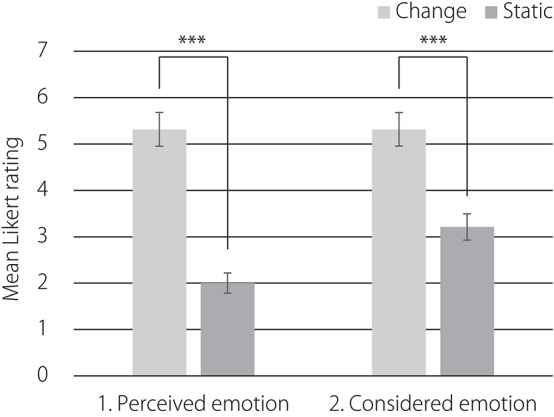
**Post-experiment questionnaire**. Error bars indicate standard errors. ^***^p < 0.001.

Figure 9 shows the results of the post-experiment questionnaire. The Brunner-Munzel test, *W* = 9.7, *p* < 0.01, revealed that the ratings for perceiving emotions from the line-drawing of a face were significantly higher in the facial expression condition than in the static face condition. The Mann–Whitney *U*-tests, *U* = 47.5, *z* = 3.95, *p* < 0.001, revealed that ratings for ratings for considering this emotion were significantly higher in the facial expression condition than in the static face condition.

### 3.3. Discussion

The results show that offers were higher in the change of facial expression than in the static face condition, confirming that emotional expression given by an online responder through an avatar face composed of simple lines led participants to behave more altruistically to the responder. The results of the post-experiment questionnaires support the behavioral result.

## 4. General discussion

The behavioral and questionnaire results for Study 2 were similar to those of Study 1, confirming that emotional expression conveyed through simple line drawings representing a robot's face has the function of eliciting altruistic behavior from humans to the same extent as human telepresence.

However, it appears that the duration of time spent deciding the offer amount for a human responder was longer than that for a robot. This indicates that those participants who played the game against a human took more time to find the point of compromise. Despite this fact, interestingly, the mean final offers were almost the same between Study 1 and Study 2. It is possible that humans have a cognitive tendency to treat robots as non-negotiable partners and that this leads to a shorter duration of time spent exploring the point of compromise. However, the facial expression of the robot might have suppressed this cognitive tendency and led the participants to be more altruistic.

The results of our studies are consistent with those of previous studies (de Melo et al., 2010, 2011). The studies of de Melo et al. (2010, 2011) and ours all showed that the emotional expressions of artificial agents are effective in inducing humans to cooperate. de Melo et al. (2010, 2011) used a human-like virtual agent, whereas we used a real robot with a simple line drawing depicting its face. This implies that sophisticated human-likeness is not necessarily needed for a cooperative relationship to develop between robots and humans. This might be because facial expressions, even the face is a line drawing, are processed subcortically (Johnson, 2005; Britton et al., 2008). Nishio et al. (2012) have conducted experiments with an android robot that has a highly human-like appearance and concluded that the appearance of the agent does not affect cooperativeness. From these results, we would suggest that the ability to interact is more important than a human-like appearance for an artificial agent to develop a cooperative relationship with a human.

A substantial number of studies have shown that in economic games played by humans, facial expressions affect the decision to cooperate or not regardless of the type of game [e.g., ultimatum games (Mussel et al., 2014), prisoner's dilemma (Reed et al., 2012), dictator games (Brown and Moore, 2002), and trust games (Tortosa et al., 2013)]. Furthermore, whereas de Melo et al. (2010) used the prisoner's dilemma, we used an ultimatum game, and both studies show that the emotional expressions of artificial agents are effective in inducing humans to cooperate. Overall, it is possible that the emotional expressions of artificial agents are useful for building cooperative relationships with humans regardless of the type of game. However, long-term field study should be conducted to investigate whether the emotional expression contributes to the initiation and maintenance of real human-robot cooperative relationships.

Some limitations occur in the present study. First, while our participants were selected from a small, culturally homogeneous population and the gender ratio was not controlled, studies have suggested that culture (Russell, 1994; Hess et al., 2000; Mandal and Ambady, 2004) and gender (Hess et al., 2000; Mussel et al., 2014) influence the expression and interpretation of emotions. Larger and more diverse samples should be used to examine gender and cultural effects on human-robot cooperative relationships mediated by emotional expressions. Second, we used a small humanoid robot, and its facial expression was shown on an LCD monitor that was mounted as its head. Further, investigation using various types of robots such as life-sized humanoid robots and robots with sophisticated facial expression mechanisms should be performed to generalize the findings.

## Author contributions

KT designed the experiments; CT performed the experiments; KT and CT analyzed the data; and KT wrote the manuscript.

### Conflict of interest statement

The authors declare that the research was conducted in the absence of any commercial or financial relationships that could be construed as a potential conflict of interest.
